# Determination of Picomolar Concentrations of Paraoxon in Human Urine by Fluorescence-Based Enzymatic Assay

**DOI:** 10.3390/s19224852

**Published:** 2019-11-07

**Authors:** Giovanni Paolo Cetrangolo, Carla Gori, Janis Rusko, Sara Terreri, Giuseppe Manco, Amelia Cimmino, Ferdinando Febbraio

**Affiliations:** 1 Institute of Biochemistry and Cellular Biology – National Research Council (CNR), via Pietro Castellino 111, 80131 Naples, Italy; g.cetrangolo@ibp.cnr.it (G.P.C.); c.gori@ibp.cnr.it (C.G.); Janis.Rusko@bior.lv (J.R.); 2 Institute of Food Safety, Animal Health and Environment “BIOR”, Lejupes street 3, LV-1076 Riga, Latvia; 3 Institute of Genetics and Biophysics “Adriano Buzzati Traverso”–National Research Council (CNR), via Pietro Castellino 111, 80131 Naples, Italy; sara.terreri@igb.cnr.it (S.T.); amelia.cimmino@igb.cnr.it (A.C.)

**Keywords:** Organophosphate pesticides, paraoxon, esterase-2, EST2, biosensor, biomonitoring, human urine, 4-methylumbelliferyl butyrate, *Alicyclobacillus acidocaldarius*, fluorescence

## Abstract

Organophosphate (OP) pesticides are widely used in the agricultural field and in the prevention of pest infestation in private and public areas of cities. Despite their unquestionable utility, several of these compounds demonstrate toxic effects to the environment and human health. In particular, the occurrence of some organophosphate pesticides is correlated to the incidence of nervous system disorders, especially in children. The detection of pesticide residues in the human body represents an important task to preserve human health. In our work we propose the use of esterase-based biosensors as a viable alternative to the expensive and time-consuming systems currently used for their detection in human fluids. Using the esterase-2 activity, coupled with a fluorescence inhibition assay, we are able to detect very low concentration levels of diethyl (4-nitrophenyl) phosphate (paraoxon) in the range of the femtomole (fmol). Method robustness tests indicate the stability of esterase-2 in a diluted solution of 4% human urine, and we are able to accurately determine concentration levels of paraoxon in the range from 0.1 to 2 picomoles (pmol). The system sensitivity for OP detection is calculated at 524 ± 14.15 fmol of paraoxon recognized at 10% of inhibition, with an estimated limit of quantification of 262 ± 8.12 pmol mL^−1^. These values are comparable with the most recent analysis methods based on mass spectrometry carried out on human samples for pesticide detection. This research represents a starting point to develop cheap and fast testing methods for a rapid screening of toxic substances in human samples.

## 1. Introduction

Human biomonitoring is one of the challenges of our century, as it is crucial to assess overall chemical and biological exposure to humans. Unlike the past, technological progress, as well as improvements in agricultural and industrial methods, increase the world’s chemical production one hundred fold, leading to an inevitable introduction of an increased amount of toxic substances in the environment. 

Unfortunately, only a small subset of these chemicals is sufficiently characterized in order to assess their toxicity [1,2]. During the last 50 years, an increased use of neurotoxic substances in several human activities was registered, the most representative in agriculture, where organophosphate (OP) pesticides replaced the more environmentally persistent organochlorine pesticides. Despite their lower resistance to degradation, OPs became the most widely used chemicals for which only recently neurotoxic effects have been reported [3,4,5,6,7]. Several authors describe a relationship between OP pesticide use and the development of neurodevelopmental disorders, particularly in children, because of high exposure levels due to lower body weights and the fact that they are a susceptible population, as their physiological detoxification systems are less efficient than adults [4,7,8,9]. Despite the effort made by the European Union (EU) to provide well-documented regulation on the use of pesticides in agricultural practice (see EU policy), pesticides are still widely spread in water and soil due to several environmental factors, such as rain and wind. For these reasons the biomonitoring of these compounds in the human body is mandatory [10]. The national report (2009–2019) on human exposure to environmental chemicals by the Centers for Disease Control and Prevention (Atlanta, GA, USA) at the National Center for Environmental Health (USA) (https://www.cdc.gov/exposurereport/), is representative of the efforts for population biomonitoring. In the last years, the technological advance of analytical systems, decreasing the limit of detection for several chemicals, allows researchers to increase the number of compounds detected, but we probably do not yet have a complete picture of the contaminants present in the human body.

The common procedures for carrying out OP detection and quantification are gas chromatography–mass spectrometry (GC-MS) and liquid chromatography–mass spectrometry (LC-MS) analytical techniques [11,12]. However, these powerful tools are becoming less convenient for this type of analysis because of their high cost, long duration of analysis, the requirement of skilled labor and delaying in the analysis by requiring sample delivery and storage.

Since biological samples could transform or even degrade some components during storage and analysis time, faster and readily-available testing methods are necessary. The development of new, selective and sensitive sensors allows for increasingly accessible and competitive ways for the biomonitoring of these toxic and hazardous substances [13,14,15,16]. Several types of biosensors for OP detection are already proposed. The most known are biosensors based on the acetylcholinesterase (AChE) inhibition [17]; however, several limitations affect these enzymes, such as the low stability to the environment and specificity toward inhibitor compounds. In our previous work, we propose the use of esterase-2 from *Alicyclobacillus acidocaldarius* (EST2) as a more efficient alternative in terms of stability (resistance to temperature, organic solvents, detergents, etc.) and specificity toward paraoxon and methyl-paraoxon [18,19,20,21]. In particular, EST2 similarly acetylcholinesterase activities show less affinity toward thio-OP, although it is able to reversibly bind many of these compounds, such as parathion and chlorpyriphos [20,21]. EST2 is a well characterized enzyme [22,23] that shows a very high affinity toward paraoxon, a very toxic OP pesticide [18], by producing a stable covalent complex that irreversibly inactivates the enzyme. Moreover, this enzyme shows a comparable efficiency of binding to paraoxon and sensitivity of inhibitor detection, allowing the detection up to 10 pmol of neurotoxic agent [19], and better selectivity than the non-specific reactions of acetylcholinesterase, the main target of OP pesticides [20]. The principle of paraoxon detection using EST2 is based on an indirect measurement method. Through the determination of residual enzyme activity, we are able to estimate the amount of enzyme that is still active (unbound to the inhibitor) by calculating the difference in moles of the inhibited enzyme. Hence, it is possible to determine the amount of paraoxon corresponding to the moles of the inhibited EST2, due to the reaction stoichiometry of EST2:paraoxon being in a ratio of 1:1, and the fact that the equilibrium of the inhibition reaction is reached within few seconds [18]. Increasing the assay sensitivity for substrate in order to measure a smaller amount of enzyme and decreasing the amount of enzyme used for the detection, resolves in an increased sensitivity toward the inhibitor concentrations and improved limit of detection.

In the present paper, we describe an improvement of the detection limits for the measurement of OP, and explore the opportunity of a new application of EST2 for the human biomonitoring of paraoxon. In particular, to increase the sensitivity threshold of OPs detection by using EST2, we investigate the use of a fluorogenic substrate such as 4-Methylumbelliferyl butyrate (4-MUBu) [24] to measure the residual enzymatic activity. Moreover, considering the fact that we are already been able to perform activity and inhibition assays of EST2 in complex solutions, such as fruit juices [19], we perform robustness tests of the enzyme stability and activity in the presence of increasing concentrations of human fluids, such as blood and urine. Finally, we evaluate the EST2 performance of paraoxon detection using this approach on human urine and blood serum, in order to propose a new fast and specific assay for the biomonitoring of these molecules in human fluids.

## 2. Materials and Methods

### 2.1. Reagents

All reagents (Sigma-Aldrich, St. Louis, MO, USA) were of analytical grade and obtained from commercial sources. 2-[4-(2 Hydroxyethyl)-1-piperazino]-ethansulfonic acid (HEPES), 4-methylumbelliferyl butyrate (4-MUBu), 4-methylumbelliferone (4-MU), diethyl (4-nitrophenyl) phosphate (paraoxon), were from Sigma-Aldrich (St. Louis, MO, USA).

### 2.2. Collection of Human Urine Samples

All samples were collected in accordance with European ethical guidelines, and those who agreed to participate in this study provided written consent. Human urine samples were collected from two healthy adult volunteers in 50 mL sterile falcons. After centrifugation at 14,000 rpm for 10 min to remove particulate matter, all urine samples were used immediately after collection in the fluorescence experiments.

### 2.3. Enzyme Purification

EST2 was over-expressed in the mesophilic host *E. coli* strain BL21 (DE3) and purified as previously described in Manco et al. [22]. The protein concentration was estimated by the optical absorbance at 280 nm, using a molar extinction coefficient of 1.34 × 10^5^ M^−1^ cm^−1^ in 40 mM sodium phosphate buffer, pH 7.1 (slightly alkali), at 25 °C, as described in Manco et al. [22].

### 2.4. MU Standard Calibration Curve in HEPES

Stock solution of 1 mM methylumbelliferone (MU) in 100 % dimethyl sulfoxide (DMSO) was prepared as a standard reference for the calculation of the reaction product concentrations in our experimental conditions. Fluorescence emission was measured at 445 nm after excitation at 365 nm for MU solution aliquots at different concentration levels in the range from 0.01 to 0.66 mM. The slopes obtained from the plotted fluorescence intensities versus the MU concentrations were used for the determination of fluorescence intensity coefficients, further used to quantify the amount of MU released during the hydrolysis of MU by EST2. All measures were carried out at least three times, and the acquired data was analyzed using the software QtiPlot 0.9.7.10 (Copyright 2004–2009 Ion Vasilief, IONDEV SRL - Bucuresti, Romania).

### 2.5. Fluorescence Standard Enzymatic Assay

The standard assay was prepared in a 0.5 mL final volume reaction mixture containing 25 mM HEPES buffer, pH 7.0 (neutral), 1% C_14_H_22_O(C_2_H_4_O)*_n_* (TRITON X100) and 1 mM 4-MUBu (from a stock solution of 40 mM 4-MUBu in 100% DMSO). After 2 min incubation at 30 °C, aliquots of EST2 (1.46 pmol) in 25 mM HEPES at pH 7.0 were added to the assay solution. 

Fluorescence measurements were carried out in a JASCO FP-777 spectrofluorimeter (Jasco Analytical Instruments, Tokyo Japan), equipped with an external thermostatic bath Julabo F25 (Julabo GmbH, Seelbach, Germany) at the temperature of 30 °C, using a quartz cuvette of 1 cm optical path. The rate of 4-MUBu hydrolysis by the EST2 enzymatic activity was determined by monitoring the increase of the fluorescence emission at 445 nm (Ex = 365 nm) due to the release of 4-MU as a reaction product. The coefficient of 4-MU fluorescence intensity, determined as previously described, was used for the calculation of the concentration of reaction products. One unit of enzymatic activity was defined as the amount of enzyme required to release 1 μmol/min of 4-MU under the indicated experimental conditions.

### 2.6. Docking Analysis

Computer simulations were carried out as described in Carullo et al. [20] using the 3D crystallographic structure of EST2 resolved at 2.6 Å (ID number 1EQV from the Protein Data Bank (http://www.rcsb.org/pdb/)). In particular, the EST2 pdb file was opportunely edited by removing the 4-(2-hydroxyethyl)-1-piperazine ethanesulfonic acid bound to the serine 155 residue in the catalytic site, and the 2-amino-2-hydroxymethyl-propane-1,3-diol molecule bound to the protein during the crystallization process. Using Avogadro software (https://avogadro.cc/), the resulting structure was optimized through a brief energy minimization, by using the Assisted Model Building with Energy Refinement (AMBER) force field with the steepest descent algorithm, in order to remove potential problems, such as bad contacts, clashes and nonphysical contacts/interactions. The 4-MUBu and p-Nitrophenyl butyrate 3D structures were generated using Avogadro software (https://avogadro.cc/) and the structures optimized through the MMFF94 force field with the steepest descent algorithm. The docking analysis was carried out using Autodock Vina, that employed the Broyden-Fletcher-Goldfarb-Shanno algorithm, significantly improving the average accuracy of the binding mode predictions with respect to AutoDock 4 [25]. A box of about 89 Angstrom^3^ was used to include both catalytic protein pockets, with an exhaustiveness of 8. During the docking procedure, both the protein and ligands are considered as rigid, because the rigidity of the double ring of 4-MUBu and of the thermophilic proteins at room temperature. The structures were analyzed and the images produced by using the PyMOL (Schrödinger - New York, NY, USA) molecular graphic software [26].

### 2.7. Kinetic Constants

Enzyme kinetic constants on 4-MUBu were determined under standard assay conditions at substrate concentrations in the range from 0.1 to 4 mM. The kinetic constant values (K_M_ and k_cat_) were calculated by plotting the reciprocals of EST2 hydrolysis rates versus the substrate concentrations (Lineweaver-Burk transformation plot). All measures were carried out at least three times, and the data were analyzed by the software QtiPlot 0.9.8.9 (Copyright 2004–2011 Ion Vasilief, IONDEV SRL - Bucuresti, Romania).

### 2.8. Inhibition Assay of EST2 in Presence of Paraoxon

10 mM stock of paraoxon in 100% DMSO was prepared in order to use as an EST2 activity inhibitor. The inhibition assays were carried out under the standard assay conditions by incubating 1.46 pmol of EST2 in presence of increasing concentrations of paraoxon in the range from 0 to 2.1 pmol in a final volume of 10 μL. After 1 min incubation, aliquots of inhibited enzyme were removed from the mixture, and the residual activity was measured in the standard assay conditions. All measures were carried out at least three times, and the data was analyzed by the software QtiPlot 0.9.8.9 (Copyright 2004–2011 Ion Vasilief, IONDEV SRL - Bucuresti, Romania). The inhibition percentage was calculated based on the equation (I_0_ – I)/I_0_ × 100, as previously described [27], in which I_0_ represents the inhibition percentage in the absence of an inhibitor, and I the percentage of inhibition at the indicated inhibitor concentration.

### 2.9. EST2 Residual Activity in Human Urine

Aliquots of a urine sample in the concentration from 0 to 10 % were added to a solution (50 μL final volume) containing 1.46 pmol of EST2 in buffer 25 mM HEPES, pH 7.0, and incubated for 5 min at 25 °C. Aliquots of 45 μL were withdrawn from the mixture, and the activity of EST2 was then measured in the standard assay conditions in the presence of 1 mM 4-MUBu. All measures were carried out at least three times, and the data were analyzed by the software QtiPlot 0.9.8.9 (Copyright 2004–2011 Ion Vasilief, IONDEV SRL - Bucuresti, Romania).

### 2.10. Paraoxon Inhibition Assay of EST2 in Human Urine

Aliquots of 1.46 pmol of EST2 were incubated in a solution (50 μL final volume) containing 4% urine in buffer 25 mM HEPES, pH 7.0, and increasing concentrations of paraoxon in the range from 0 to 2.1 pmol. After 1 min incubation, aliquots of 45 μL were removed from the mixture and the EST2 residual activity was measured in the standard assay conditions. All measures were carried out at least three times, and the data were analyzed by the software QtiPlot 0.9.8.9 (Copyright 2004–2011 Ion Vasilief, IONDEV SRL - Bucuresti, Romania).

## 3. Results and Discussion

### 3.1. 4-MUBu Fluorescence Measurements and the Determination of Kinetics Parameters

**Scheme 1:**
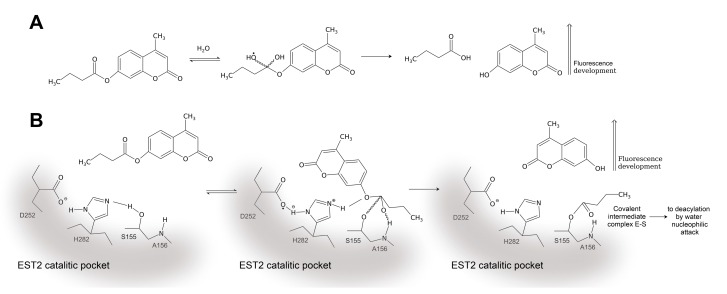
Mechanism of (**A**) the spontaneous hydrolysis of 4-methylumbelliferyl butyrate (4-MUBu), in 2-[4-(2 Hydroxyethyl)-1-piperazino]-ethansulfonic acid (HEPES) buffer and (**B**) by enzymatic hydrolysis after the addition of esterase-2 (EST2). The release of 4-methylumbelliferone (4-MU) produces the increase in fluorescence.

**Figure 1:**
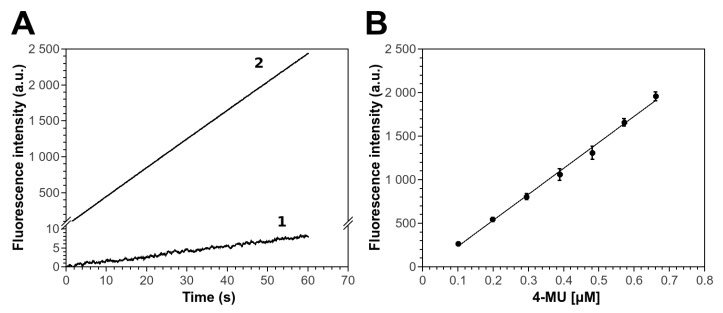
EST2 assay using 4-MUBu. (**A**) Fluorescence development of 4-MUBu by spontaneous hydrolysis in HEPES buffer (curve 1) and enzymatic hydrolysis after the addition of EST2 (curve 2). (**B**) Calibration curve of fluorescence intensity at increasing concentration of 4-MU in HEPES buffer for the calculation of the coefficient value necessary for the determination of the amount of reaction products from the enzymatic hydrolysis (R square value for the regression line = 0.996). The error bars represent the standard deviation (SD) from three independent experiments.

The sensitivity of fluorescence detection is approximately one thousand times greater than absorption spectrophotometric methods. This leads to greater limits of detection while potentially using less sample material. In order to develop detection methods able to discriminate smaller amounts of organophosphate (OP) inhibitors, we set up the conditions for the measurements of EST2 activity using 4-MUBu as a fluorogenic substrate. One of the first uses of 4-MUBu as fluorogenic substrate was described in 1977 to easily and rapidly detect mycobacterial esterase [28]. In particular, the use of this substrate allows detecting very low lipolytic activity [24], while testing multiple samples simultaneously and maintaining short incubation times. We observe a suitable resistance of 4-MUBu to the spontaneous hydrolysis (Scheme 1A) at neutral pH, using 25 mM HEPES pH 7.0 (curve 1 in Figure 1A), a zwitterionic organic chemical buffer, so that all experiments are carried out in these conditions. In agreement with an enzyme-substrate reaction, as described in the Scheme 1B, we observe a linear increase of the signal of fluorescence intensity after the addition of EST2 to the 4-MUBu solution, for the release of 4-MU (curve 2 in Figure 1A). A coefficient value of 57.16 ± 1.03 fluorescence units for pmol of 4-MU, is determined by using a calibration curve in HEPES (Figure 1B), allowing the determination of the amount of the reaction products. Measuring the amount of 4-MU released by EST2 activity at different 4-MUBu concentrations, we calculate the values of kinetic constants with this substrate. The K_M_ and k_cat_ values of 221.6 μM and 30.7 × 10^2^ s^−1^, respectively, are determined for the hydrolysis of 4-MUBu by measuring the EST2 activity at 30 °C in 25 mM HEPES buffer pH 7.0. 

We compare the EST2 kinetic constants determined by using 4-MUBu as a substrate, with the ones determined on p-Nitrophenyl butyrate, a chromogenic substrate having the same carbon chain length of the acidic moiety. The values obtained on fluorogenic substrate differ about 2-fold with respect to the kinetic constants previously determined using p-Nitrophenyl butyrate as substrate in 40 mM sodium phosphate pH 7.0 at 70 °C (90 ± 6 μM and 18.5 ± 0.9 × 10^2^ s^-1^) [29]. The different affinity could be easily explained having used an assay temperature of 40 °C lower than the optimal one. This makes the protein structure more rigid to the binding, with the substrate generally decreasing the enzyme affinity. However, the enzyme characterization at 30 °C is justified for its use at room temperature as a biosensing device. Moreover, the greater steric hindrance of the two phenolic rings of 4-MUBu with respect to the single ring of p-nitrophenyl ester in the chromogenic substrates could play a different role in the binding at the active site of the enzyme.

**Figure 2:**
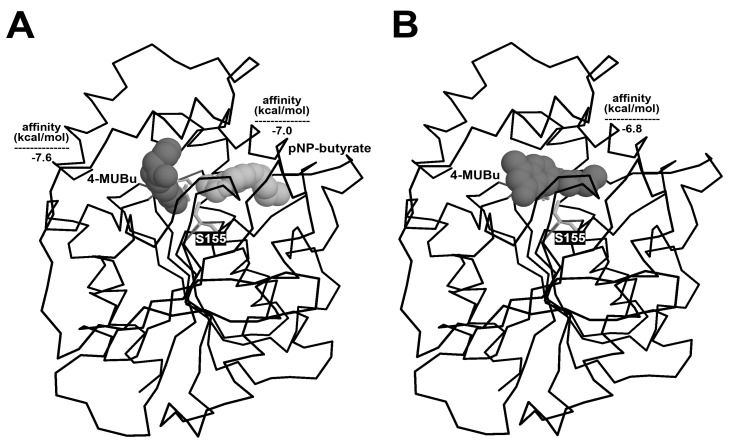
Docking analysis with binding free energies (shown as kcal mol^-1^) of complexes between EST2 and substrates. Trace representation of EST2 backbone (black), residues in the catalytic site are represented as stick in gray, the substrates MUBu (dark gray) and pNitrophenyl-butyrate (gray) are depicted as a van der Waals surface (WDV) representation. (**A**) Accommodation of 4-MUBu in the alcohol pocket of EST2, with respect the pNitrophenyl-butyrate bonding to the acyl one; and (**B**) arrangement of 4-MUBu in the catalytic site with less affinity with respect to the alcohol pocket.

Notably, the poses with the lowest energy of binding, or binding affinity, determined for both substrates by docking simulations, highlights unexpected accommodation of 4-MUBu in the alcohol-binding site of the enzyme, differently from the p-Nitrophenyl butyrate which is accommodated in the acyl pocket one (Figure 2A). 

This result is in accordance with the substrate-induced switch demonstrated for long chain substrates (with high steric hindrance) in the EST2 reaction mechanism [23]. Further, less binding free energy (about 1 kcal/mol), with a distance from the best pose determined by the root-mean-square deviation (RMSD) lower and upper bound of 4.326 and 7.494, respectively, is measured for the binding of the two phenolic rings of 4-MUBu into the acyl-binding pocket of the EST2 catalytic site (Figure 2B). The energy released in the formation of noncovalent bonds is only 1−5 kcal/mol, much less than the bond energies of single covalent bonds; moreover, the average kinetic energy of molecules at room temperature (25 °C) is about 0.6 kcal/mol. Considering the energies involved in these interactions, the allocation in the alcohol pocket is favorite with respect to the acyl one. Despite the different affinity shown towards the fluorescent substrate by EST2, we almost double its efficiency of catalysis, and most importantly, the assay sensitivity for substrate is improved about 20 times. In fact, at room temperature, using colorimetric substrates, we are able to measure a stable signal for the enzymatic activity, assaying an amount of enzyme of about 29.2 pmol [19]. Performing fluorescence assays in similar conditions of pH and temperature (30 °C), we are able to efficiently measure with high reproducibility and repeatability the enzymatic activity of about 1.46 pmol of EST2.

### 3.2. EST2 Inhibition with Paraoxon Determined by Fluorescence Assay

**Figure 3:**
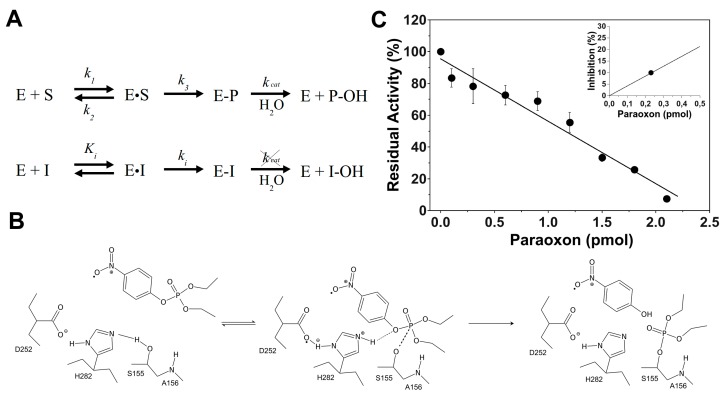
Inhibition of EST2 by diethyl (4-nitrophenyl) phosphate (paraoxon). Description of the kinetic (**A**) and molecular (**B**) mechanism of the inhibition of EST2 by paraoxon. The inhibited enzyme is frozen in a stable covalent intermediate with the organophosphate group of pesticide. (**C**) Plot of EST2 residual activity in presence of an increasing concentration of paraoxon. In the insert, the percentage of the inhibition of EST2 in the presence of this increasing concentration of paraoxon. The error bars represent the standard deviation (SD) from three independent experiments.

The EST2 inhibition mechanism is proposed in our previous work [18], taking into account the widely described reaction mechanism [23] (Figure 3A). In summary, the affinity constant towards the inhibitor (K_i_) represents the K_M_ constant for the substrate, while the inhibition rate constant k_i_ corresponds to the acylation constant k_2_. The rate constant of deacylation (k_cat_), very high in the enzymatic reaction on the substrate, becomes very small in the irreversible inhibition kinetics, freezing the enzyme-inhibitor intermediately into a very stable complex (Figure 3A).

The EST2 affinity towards paraoxon results higher than synthetic substrates, being that the K_i_ is not determinable by using the conventional method of pseudo-first-order kinetics [18] for the determination of irreversible inhibition constants. Then the formation of the covalent bond between the side-chain of serine residue 155, in the catalytic site, and the reactive organophosphate group of paraoxon [18], is favored (Figure 3B). 

This covalent intermediate results in a very stable conformation that irreversibly inhibits the enzyme, requiring a stronger nucleophilic group, with respect to water, in order to complete the next deacylation step to release the free-enzyme [18,19]. In particular, the balance of this reaction is completely shifted towards the formation of the covalent intermediate, in a matter of few seconds, obtaining the full inhibition of an amount of enzyme corresponding to the quantity of paraoxon added in a concentration ratio 1:1 [18,19]. Taking advantage of the high affinity of EST2 towards these compounds allows us to utilize the residual activity of the enzyme after irreversible inhibition for the fast detection and quantification of OP in human and environmental samples. The fluorometric assay allows us to measure the EST2 residual activity in the presence of very low concentration levels of paraoxon in the range from 100 to 2100 fmol (Figure 3C). The equation of linear regression which is determined for the residual activity against the paraoxon concentrations shows the R^2^ > 0.96, indicating a linear response for the determination of the residual activity in this range of concentrations. 

The calculation of the percentage of inhibition, after incubation with paraoxon in our experimental conditions, permits us to calculate a value of 230 fmol for the tested pesticide at a 10% of inhibition (insert in Figure 3C). Compared to our previous work [19], we improve the limit of detection for paraoxon using EST2 of about 80-fold, reaching a quantification limit of 125 fmol. In this way, we demonstrate a potential of detection abilities comparable to the more sophisticated analytical systems recently used.

### 3.3. Paraoxon Determination in Human Samples

**Figure 4:**
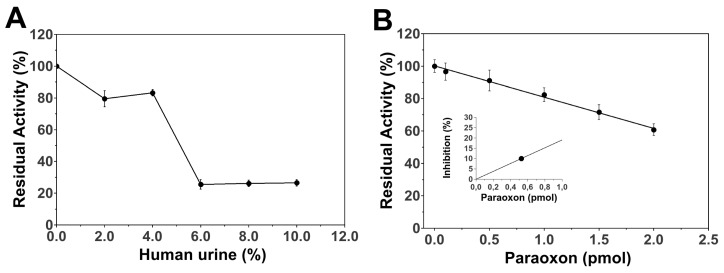
Determination of paraoxon in human urine. (**A**) Plot of EST2 residual activity in presence of increased amount of urine. (**B**) Plot of EST2 residual activity in 4% urine, in the presence of the increasing concentration of paraoxon. In the insert, the percentage of inhibition of EST2 in the presence of this increasing concentration of paraoxon. The error bars represent the SD from three independent experiments.

The main problem related to the use of a substrate for esterase activity is represented by the presence of lipase activities in human fluids. In fact, the measurements carried out on assay solutions containing serum aliquots from human blood (see Appendix A) highlight that is it impossible to measure the EST2 activity due to the massive presence of lipase activities in the blood, which hydrolyzes the 4-MUBu. New methodologies need to be developed in order to selectively inhibit the lipase activities, using inhibitors inactive on EST2, or removing them from the samples by a preliminary protein aggregation/precipitation step, that allows for the measurement of EST2 residual activity in human serum for the detection of OP metabolites in blood. Instead, the low amount of lipase activities, less than 1 unit/dL in 24-h urine samples from healthy donors, has made it possible to measure the EST2 activity in samples of this human fluid. In order to verify if urine affects the EST2 activity, we measure the enzymatic activity at increasing concentrations of urine (Figure 4A). 

EST2 activity is affected by the addition of urine concentrations higher than 4%, so we use this as an appropriate final concentration for the inhibition measurements. We measure a decrease of enzymatic activity after EST2 incubation in the presence of a mixture containing 4 % urine and paraoxon (Figure 4B). However, the enzyme does not appear to be completely inhibited by a stoichiometric amount of paraoxon, in agreement with our previous data on complex solutions, such as fruit juices [19]. Probably, as described in the irreversible inhibition kinetics [18], the retained enzyme activity could be explained by the presence of protecting substances that compete with the irreversible inhibitor for the same binding site. Since human urine is a complex solution that reflects the physiological state of an individual, we cannot exclude that in some samples, i.e., from individuals following specific diets or under medication, there could be chemicals present which could regenerate the enzyme activity after inhibition or affect the fluorescent emission of 4-MU (by fluorescence quenching). 

Exploiting the high specificity of EST2 towards paraoxon, the improved sensitivity of the fluorescence assay on the substrate and the low amount of urine (only 4%) required for the measurement, we can reduce the effects coming from the presence of unwanted metabolites in urine samples. Moreover, in order to completely solve the exposed problems, two fast reference tests (quality control samples) could be included in the measurement in order to determine the 100% activity in presence of the urine sample, and the residual activity at a known paraoxon concentration. The equation of linear regression determined for the residual activity against the paraoxon concentrations shows R^2^ > 0.99, indicating a good linear response for the determination of the residual activity in the presence of 4% urine. From our measurements we calculate an amount of 524 ± 14.15 fmol of paraoxon recognized at 10% of inhibition, with a limit of quantification of 262 ± 8.12 pmol mL^-1^ (insert Figure 4B). These results are comparable to the values obtained in similar matrices by novel mass spectrometry detection methodologies. Dulaurent et al. describes a pesticide multi-method employing the LTQ linear ion trap apparatus, in which the quantification limits reach 36 pmol mL^-1^ of paraoxon in blood and the possibility of similar detection limits in other bodily fluids [30]. These values were comparable to the amount determined by LC and GC-MS in urine samples of donors exposed to OPs [31].

## 4. Conclusions

The detection of chemicals in the human body represent an important task to preserve human health. At the current time, the biomonitoring of human exposure to environmental chemicals is carried out by measuring the substance occurrence levels in people’s blood and urine (https://www.cdc.gov/exposurereport/), reflecting the overall chemical exposure. In order to determine which substances, their corresponding concentration levels and the proportion of the population associated with adverse health effects, a high number of samples from a large cohort of participants is required. This means that an extensive analysis must be performed in a short amount of time in order to provide the results as fast as possible, preferably at a low cost. Over time, some substance concentration levels measured in the samples could change. Especially in the scenarios of acute contamination events, it is important to perform the analysis as fast as possible, due to the rapid toxicological effects and biotransformation of the substances *in vivo*. The methods for the detection of pesticides and other compounds of concern, such as GC- and LC-MS techniques, have some limitations with respect these requirements. Faced with this situation, biosensors could represent a future opportunity for the fast detection of chemicals in human fluids, and distinctively, the pesticides that are widely spread throughout the environment. 

In our research we demonstrate the potential use of EST2 as a bioreceptor for a biosensing device for the detection of paraoxon in urine. The use of a fluorescent substrate increases the sensitivity of the assay several times, allowing us to measure the activity of very low amounts of enzyme (pmol). This result translates into the possibility of measuring fmol of paraoxon that binds to EST2. Moreover, the enzyme maintains a sufficient residual activity in a complex solution like urine, although a dilution of the original sample is required. In conclusion, utilizing the stability, high activity, low production and purification costs as the characteristics of this enzyme [18,19,20,21,22], a simple and cheap assay kit for OP compounds, similar to existing analysis kits for glucose, could be easily developed. Moreover, due to the high affinity for these substances, these types of biosensing devices could allow for the detection with minimized reference testing, thus improving the simplicity of assays and the time of detection. Further research is ongoing to implement the recognition of a larger number of substances, such as parathion, chlorpyrifos and other OP derivatives, with good efficiency for the development of commercially-available biosensing devices.

## Appendix A

- Collection of Human Blood Samples

All samples were collected in accordance with European ethical guidelines, and those who agreed to participate in this study provided written consent. Human blood samples of 10 mL were collected from two healthy adult volunteers in a sterile tube. After 30 min to allow the blood to clot, serum was separated by centrifugation at 2000 rpm for 10 min and stored at 4 °C to be used in the fluorescence experiments.

- EST2 Residual Activity in Human Blood

Aliquots of blood serum from 0 to 10% were added to a solution (50 μL final volume) containing 1.46 pmol of EST2 in buffer 25 mM HEPES, pH 7.0, and incubated for 5 min at 25 °C. Aliquots of 45 μL were withdrawn from the mixture, and the activity of EST2 was measured in the standard assay conditions in the presence of 1 mM 4-MUBu.

- Inhibition Assay of EST2 in Human Blood

Aliquots of 1.46 pmol of EST2 were incubated in a solution (50 μL final volume) containing 4% blood in buffer 25 mM HEPES, at pH 7.0, and increasing concentrations of paraoxon in the range from 0 to 2.1 pmol. After 1 min incubation, aliquots of 45 μL were removed from the mixture and the EST2 residual activity was measured in the standard assay conditions.
